# Linked color imaging application for improving the endoscopic diagnosis accuracy: a pilot study

**DOI:** 10.1038/srep33473

**Published:** 2016-09-19

**Authors:** Xiaotian Sun, Tenghui Dong, Yiliang Bi, Min Min, Wei Shen, Yang Xu, Yan Liu

**Affiliations:** 1 Department of Gastroenterology, the 307 Hospital of Academy of Military Medical Science, Beijing 100171, China; 2 Department of Internal Medicine, Clinic of August First Film Studio, Beijing 100161, China

## Abstract

Endoscopy has been widely used in diagnosing gastrointestinal mucosal lesions. However, there are still lack of objective endoscopic criteria. Linked color imaging (LCI) is newly developed endoscopic technique which enhances color contrast. Thus, we investigated the clinical application of LCI and further analyzed pixel brightness for RGB color model. All the lesions were observed by white light endoscopy (WLE), LCI and blue laser imaging (BLI). Matlab software was used to calculate pixel brightness for red (R), green (G) and blue color (B). Of the endoscopic images for lesions, LCI had significantly higher R compared with BLI but higher G compared with WLE (all P < 0.05). R/(G + B) was significantly different among 3 techniques and qualified as a composite LCI marker. Our correlation analysis of endoscopic diagnosis with pathology revealed that LCI was quite consistent with pathological diagnosis (P = 0.000) and the color could predict certain kinds of lesions. ROC curve demonstrated at the cutoff of R/(G+B) = 0.646, the area under curve was 0.646, and the sensitivity and specificity was 0.514 and 0.773. Taken together, LCI could improve efficiency and accuracy of diagnosing gastrointestinal mucosal lesions and benefit target biopsy. R/(G + B) based on pixel brightness may be introduced as a objective criterion for evaluating endoscopic images.

Gastrointestinal endoscopy has been widely applied for detecting and treating mucosal and submucosal lesions in gastrointestinal tract^1^,^2^. However, traditional endoscopy is not quite efficient and accurate due to either the insufficient light or the inability to observe the microstructure. Then many new endoscopic technique, such as magnified imaging and image-enhanced endoscopic system has been developed, which have been proved to improve the diagnostic accuracy^3^. Blue laser imaging (BLI) could both be used for examining the microvessels and mucosa^4^,^5^,^6^. Although BLI-bright mode has brighter endoscopic views, the imaging is still criticized for darkness, which contributes to the low diagnostic rate. Thus, linked color imaging (LCI), a newly modified endoscopic system, has been introduced. LCI images is bright and the red color has been adjusted to make the lesions be easily identified under endoscopy. Previous study has reported that the difference on the hue of the endoscopic images could be enhanced by LCI technique, which may make the red regions redder and white region whiter^7^,^8^,^9^. These results also indicated that the specific hue in LCI might be correlated certain kinds of lesions.

In order to obtain accurate diagnosis, effective endoscopic criteria that can be quantifiable are being urgently needed, which could produce relatively objective diagnosis. The characteristics of the hue in endoscopic images might be one of these candidates available like pixel brightness for RGB (red green and blue) color model, which have been rarely evaluated^3^. Our study aimed at investigating whether LCI application could improve the diagnostic accuracy of the endoscopy and further evaluating the characteristics of the hue in LCI images, which could help validate a new endoscopic examination in clinical practice.

## Results

### Demographical and clinical characteristics

In our cohort, 37 patients underwent gastroduodenoscopy and 17 patients underwent colonoscopy. Of patients for gastroduodenoscopy, the mean age was 54.7 ± 14.2 years old and the gender ratio was 2.08:1 (male: female). The main clinical manifestations included stomach pain (n = 30, 81.1%), abdominal distension (n = 22, 59.5%) and heartburn (n = 5, 23.5%). Of patients for colonoscopy, the mean age was 49.2 ± 17.1 years old and the gender ratio was 3.25:1 (male: female). The main clinical manifestations included diarrhea (n = 10, 58.9%), hematochezia (n = 7, 41.2%) and constipation (n = 6, 35.3%). There were 10 patients who underwent screening endoscopic examination. All the lesions were pathologically diagnosed, and chronic gastritis and ulcerative colitis were most commonly diagnosed by gastroduodenoscopy and colonoscopy, respectively (Table 1).

### Pixel brightness of LCI images was different from that of WLE and BLI images

**Figure 1:**
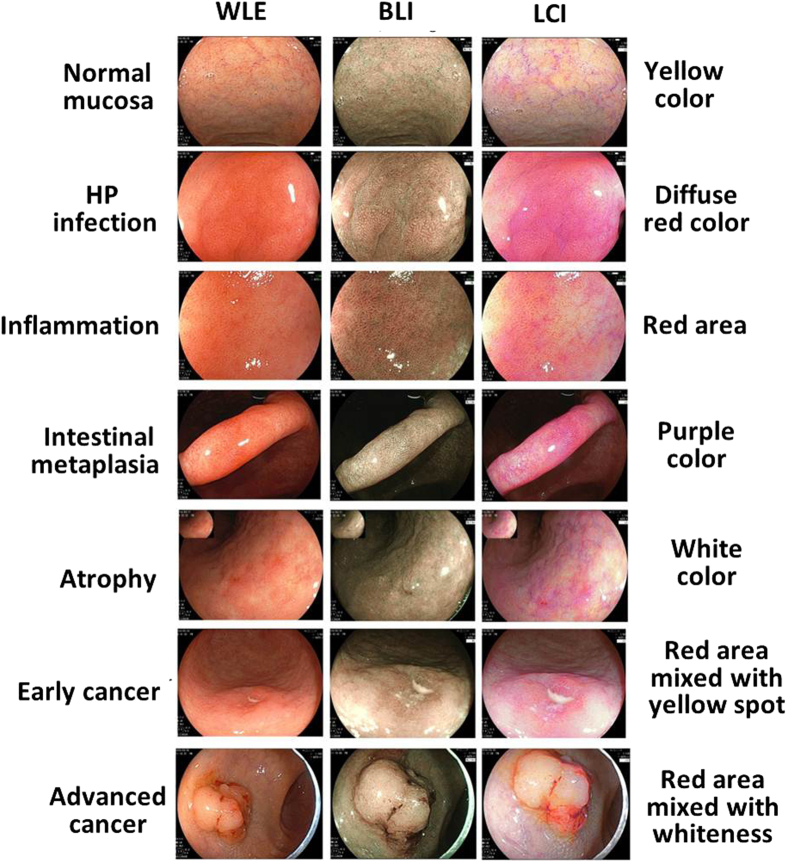
Typical endoscopic images for normal mucosa, HP infection, inflammation, intestinal metaplasia, atrophy, early cancer and advanced cancer.

**Figure 2:**
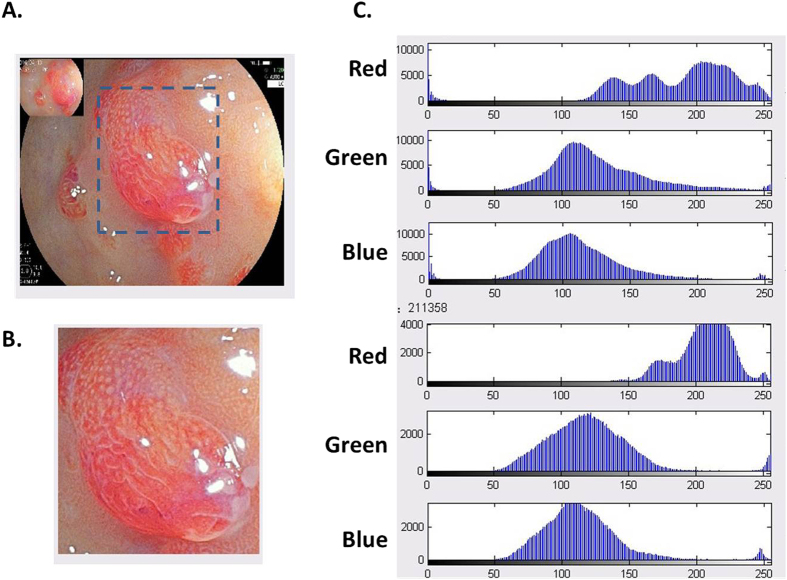
Analysis of typical endoscopic images from one patient with gastric adenomatous polyps. The area of interest in endoscopic images was selected (dashed line) and analyzed by Metalab software to calculate the pixel brightness for red, green and blue color.

In order to deepen the current understanding on the properties of endoscopic imaging, we analyzed the specific color of the images by calculating the pixel brightness for red, blue and green color, respectively (Figs 1 and 2). A total of 44 paired WLE, LCI and BLI images for the same lesion or normal mucosa were selected. Our results demonstrated that R, G and B of LCI images were higher than those of WLE and BLI images (all P < 0.05) for normal mucosa, which could validate the image enhancement by BLI and LCI technique. R of LCI images for the lesion was higher than that of BLI images (202.973 ± 26.348 vs. 198.329 ± 25.376, P = 0.000), and G of LCI images was higher than that of WLE images (128.360 ± 24.553 vs. 104.605 ± 17.974, P = 0.000), which was consistent with the design of BLI and LCI^9^. Although B of LCI images was significantly different from that of WLE and BLI images, B for normal mucosa was insignificantly from that for the lesion in all 3 endoscopic modes. Thus, we finally introduced the value of R/(G+B) as a composite marker for evaluating the endoscopic images, which was remarkably different (LCI vs. WLE, P = 0.000; LCI vs. BLI, P = 0.002) (Table 2).

### LCI imaging could facilitate the target biopsy under endoscopy

The clinical application of LCI imaging were further expanded. We correlated the endoscopic diagnosis with the pathological analysis and it was proved that LCI was most consistent with the pathology (BLI vs. WLE P = 0.029; LCI vs. WLE P = 0.000; Table 3). These results indicated that endoscopic LCI imaging could improve the efficiency and accuracy of target biopsy, which will definitely help avoid the misdiagnosis. The main color characteristics of certain lesions by LCI were also summarized (Fig. 1). Lesions of H. pylori (HP) infection were in diffusion red color (n = 20), of inflammation or cancer were in red color (n = 33), of intestinal metaplasia were in purple color (n = 21), of atrophy were in white color (n = 11) and normal mucosa were manifested in yellow color (n = 20). These data support that specific color feature of LCI images might be closely correlated with the pathology.

### Certain pixel brightness of LCI images could differentiate the lesions from normal mucosa

**Figure 3:**
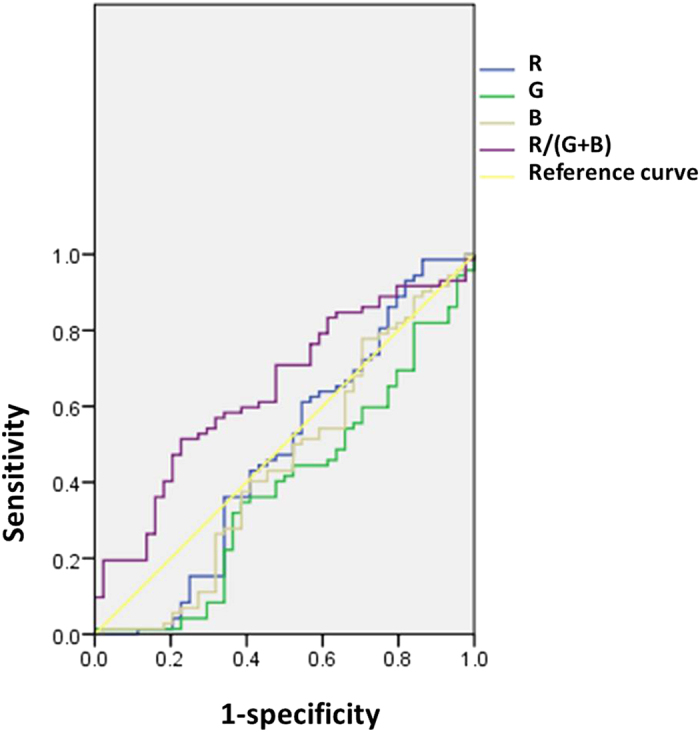
ROC curve for differentiating the lesions from normal mucosa by pixel brightness.

Although the endoscopy is useful in diagnosing mucosal lesion of gastrointestinal tract, most of the lesions were subjectively identified and there was still no objective judgment on the specific endoscopic images. Thus, based on our data, we selected R/(G + B) as a potential marker for endoscopically differentiating the lesions from normal mucosa. A total of 116 LCI images were extracted from our computerized database. ROC curve for R, G, B and R/(G + B) was drawn, respectively (Fig. 3). The area under curve for R/(G+B) was 0.646 (P = 0.008). At the cutoff of 0.8020, the sensitivity and specificity was 0.514 and 0.773 (Table 4). Taken together, pixel brightness might serve as a new method to analyze the features of the endoscopic images for proper diagnosis, especially for chromoendoscopy which is focused on the color change.

## Discussion

Great progress has been made on the endoscopic technique in order to improve the diagnostic accuracy and therapeutic efficacy^10^,^11^. Among them, chromoendoscopy and magnified endoscopy have the greatest contribution, which could help identify the lesions and observe the mucosa’s microstructure^12^. The development of LCI was based on BLI technique, which is a chromoendoscopic technique via a specially designed laser system^7^. LCI is designed to be able to obtain a sufficient brightness and easily differentiated contrast in hue. Furthermore, LCI and BLI put more emphasis on the color change of the mucosa. Our study evaluated the clinical application of LCI in diagnosing gastrointestinal mucosal lesions and the main characteristics of the LCI images were summarized for the first time, which will definitely optimize the current clinical strategy for such patients in future.

There is no doubt that the endoscopic diagnosis has been subjectively made by the endoscopists with quite limited objectivity^13^. In addition, no efficient and accurate endoscopic criteria or markers have been proposed by previous studies^10^,^14^,^15^. Thus, we used the value of pixel brightness in RGB color model to evaluate the color of the endoscopic images and further analyzed its correlations with certain clinical data. RGB color model is an additive model of a series of colors that could be reproduced by red, green and blue light in different arrangements. Pixel brightness is a physical unit in a image, which is widely used for analyzing digital imaging as a continuous variable, but pixel brightness for RGB model has not been ever reported in evaluating endoscopic images^16^,^17^,^18^. Thus, we hypothesized that pixel brightness for RGB color may be applied to judging the endoscopic images, which is of translational significance. Our results found that LCI images for the lesions had higher R than BLI but lower G compared with WLE, which could be explained by the imaging principle of LCI. LCI was BLI with red light added, and thus green and blue color in BLI will be purple and yellow color, respectively. Since the B for the lesion was insignificantly different from that for normal mucosa observed by WLE, LCI and BLI, B was excluded for further analysis. The wavelength for green (577–492 nm) and blue light (450–435 nm) is approaching, while the wavelength for red light is 760–622 nm. The wavelength determines the properties of certain light including the ability of tissue penetration. The longer the light wavelength, the stronger the tissue penetration^19^,^20^. Seen from this, the value of R/(G + B) is a potential composite marker for investigating the physical properties of WLE, LCI and BLI technique, which was also supported by our findings that R/(G + B) of LCI images for the lesions were greatly higher than those of WLE and BLI, respectively (P < 0.05). These data also indicated that the calculation of pixel brightness for RGB color may be a useful method of evaluating the endoscopic images.

Target biopsy and real-time observation has been highly recommended in recent years^21^,^22^. We compared the diagnostic accuracy of WLE, LCI and BLI using the pathology as the gold standard. It was observed that LCI endoscopic diagnosis was significantly correlated with the pathology (P = 0.000), and different lesions possessed specific color features. In our cohort, it was also supposed that the color of LCI images could be also used for evaluating the tumor infiltration. Lesions of early cancer usually were presented as red area with yellow color, while those of advanced cancer were as red area with white color. Furthermore, we focused on the identification of a quantifiable marker for obtaining endoscopic diagnosis. ROC curve for differentiating the lesion from normal mucosa was calculated and it revealed that R/(G + B) may be a potential endoscopic marker. The area under the curve was not quite large with medium sensitivity and specificity, which may be associated with the fact that the endoscopic images are manually recorded and thus exposure delay and a small shake may impair the quality of the images. Our study could establish a new computerized endoscopic diagnostic model as Miyaki *et al*. proposed^23^, which could enlighten the future research.

Our study also has limitations. First, this pilot study was a retrospective analysis and clinical data were retrieved from our electronic database. There were 10 patients who were excluded due to incomplete information. Second, the sample size was not quite large and only 44 patients were enrolled. However, as a pilot study, our results could be able to provide relatively reliable evidence for elaborating the valuable clinical usage of LCI in gastrointestinal endoscopic examinations. Third, our study mainly focused on the role of RGB color pixel brightness and other factors associated with color enhancement have not been fully examined. A multicenter large-scale prospective investigation on the clinical application of LCI technique will be conducted soon.

Gastrointestinal endoscopy has incomparable advantages over other diagnostic examinations like ultrasound and radiological images^24^. The development of LCI may be a new milestone in the history of endoscopy, which could guarantee relatively high diagnostic efficiency and accuracy by modulating the color of the imaging. We also demonstrated that pixel brightness for RGB color model could potentially quantifying the endoscopic images as a objective marker, which will be validated in future investigations. New quantifiable endoscopic criteria for diagnosing certain mucosal lesions should be explored.

## Methods

### Patients

A total number of 44 consecutive patients who had indications for diagnostic endoscopic examination were enrolled in the 307 Hospital of Academy of Military Medical Science from February 2016 to June 2016. Demographic (i.e. age, gender) and clinicopathological characteristics (i.e. main symptom, the treatment) were retrieved from the computerized database. All the patients signed the written informed consent and our study was approved by the Ethic Committee of the 307 Hospital of Academy of Military Medical Science in accordance with Declaration of Helsinki. All the methods were performed following the relevant guidelines and regulations.

### Endoscopy and LCI

All the endoscopic examinations were completed by experienced endoscopists. The indications were listed in Table 1. EC-L590ZW endoscope with the LASEREO system (FUJIFILM Co., Tokyo, Japan) was used and all the endoscopic procedures were performed routinely. No patients were under anesthesia. There were 17 patients who underwent colonoscopy and 37 who underwent gastroduodenoscopy. During the endoscopic examination, all the lesions and surrounding normal mucosa were observed by white light (WLE), LCI and BLI in turns and typical images were recorded, respectively (Fig. 1). Biopsy was performed for each lesion and the pathological diagnosis were collected. If more than 1 lesion was detected, the most severe one was included for further analysis.

### Image analysis

MatLab software (USA) was applied to analyze the WLE, LCI and BLI images as the previous study described^25^. The brightness of all the endoscopic images has been set to be comparable. The area of interest in the endoscopic images was selected, and the pixel brightness for red (R), green (G) and blue (B) color was evaluated (Fig. 2). The value of R/(G + B) was also introduced as a composite marker for comprehensive analysis.

### Statistical analysis

All the statistical analysis were performed using SPSS 17.0 software. Continuous and categorical data were shown as mean ± standard deviation (SD) and percentage (%), respectively. The differences on continuous data were tested by student t-test and on categorical data by chi-square test. Receiver operating characteristic (ROC) curve of R, G, B and R/(G + B) for differentiating the lesion from normal mucosa was analyzed and the area under curve, cutoff, sensitivity and specificity were also calculated. Two-tailed P value less than 0.05 was considered as statistically significant.

## Additional Information

**How to cite this article**: Sun, X. *et al*. Linked color imaging application for improving the endoscopic diagnosis accuracy: a pilot study. *Sci. Rep.* **6**, 33473; doi: 10.1038/srep33473 (2016).

## Figures and Tables

**Table 1:** Demographic and clinical characteristics.

	Patients who underwent gastroduodenoscopy (n = 37)	Patients who underwent colonoscopy (n = 17)	
Age, years	54.7 ± 14.2	49.2 ± 17.1	
Gender, n (%)			
Male	25 (67.6)	13 (76.5)	
Female	12 (32.4)	4 (23.5)	
Clinical manifestations, n (%)			
Stomach pain	30 (81.1)	Diarrhea	10 (58.9)
Abdominal distension	22 (59.5)	Hematochezia	7 (41.2)
Heartburn	5 (23.5)	Constipation	6 (35.3)
Screening	6 (16.2)	Screening	4 (23.5)
Pathological diagnosis, n (%)			
Chronic gastritis	26 (70.3)	Ulcerative colitis	5 (29.4)
Gastric polyp	4 (10.8)	Colon cancer	1 (5.9)
Gastrointestinal stromal tumor	1 (2.7)	Graft-versus-host disease	1 (5.9)
Mucosa-associated lymphoid tissue lymphoma	2 (5.4)	Colon polyp	7 (41.2)
Gastric ulcer	2 (5.4)	Rectum carcinoid	1 (5.9)
Gastric cancer	2 (5.4)	Normal colon tissue	2 (11.8)

**Table 2:** Pixel brightness for red, green and blue color was calculated for WLE, BLI and LCI images.

Pixel brightness	Location	WLE (n = 44)	BLI (n = 44)	LCI (n = 44)	P value for LCI vs. WLE	P value for LCI vs. BLI
Red color (R)	Lesion	198.329 ± 25.376	144.438 ± 33.109	202.973 ± 26.348	0.261	0.000
	Normal mucosa	182.403 ± 44.221	149.577 ± 48.509	199.354 ± 35.644	0.001	0.000
Green color (G)	Lesion	104.605 ± 17.974	141.644 ± 194.993	128.36 ± 24.553	0.000	0.646
	Normal mucosa	104.961 ± 26.975	115.971 ± 40.339	137.221 ± 32.581	0.000	0.001
Blue color (B)	Lesion	74.726 ± 16.721	87.505 ± 30.214	126.630 ± 23.568	0.000	0.000
	Normal mucosa	75.952 ± 22.754	87.511 ± 35.963	129.045 ± 30.562	0.000	0.000
R/(G + B)	Lesion	1.134 ± 0.196	0.731 ± 0.148	0.812 ± 0.140	0.000	0.002
	Normal mucosa	1.027 ± 0.150	0.758 ± 0.128	0.760 ± 0.093	0.000	0.889

**Table 3:** The LCI imaging could benefit the endoscopic target biopsy.

Endoscopic diagnosis	Consistence with pathology, n (%)	Inconsistence with pathology, n	P value (vs. WLE)
WLE (n = 44)	20 (45.5)	24 (54.5)	—
BLI (n = 44)	29 (65.9)	15 (34.1)	**0.020**
LCI (n = 44)	42 (95.5)	2 (4.5)	**0.000**

**Table 4:** ROC analysis on the pixel brightness of LCI images for differentiating lesions from normal mucosa.

Pixel brightness	Area under curve	Cutoff	Sensitivity	Specificity
R	0.478	—	—	—
G	0.384	—	—	—
B	0.446	—	—	—
R/(G+B)	0.646	0.8020	0.514	0.773
